# A Hybrid Giza Pyramids Construction–Crow Search Algorithm–Particle Swarm Optimization (HGPC-CSA-PSO) Framework for Simulating Dynamic Collaborative Grouping in Interpreting Education: A Simulation-Based Exploratory Study

**DOI:** 10.3390/biomimetics11090650

**Published:** 2026-09-09

**Authors:** Juan Yu, Ping Li, Xi Hu

**Affiliations:** 1 School of International Communications, Wuhan Business University, Wuhan 430056, China; 20150635@wbu.edu.cn; 2 School of Artificial Intelligence, Jianghan University, Wuhan 430056, China; huxi@jhun.edu.cn; 3 Artificial Intelligence Institute, Jianghan University, Wuhan 430056, China

**Keywords:** biomimetic optimization, swarm intelligence, HGPC-CSA-PSO, interpreting education, multi-agent modeling, knowledge diffusion, Complex Adaptive Systems, simulation-based study

## Abstract

This simulation-based exploratory study examines HGPC-CSA-PSO, a hybrid biomimetic optimization framework integrating the Giza Pyramids Construction Algorithm (GPC), Crow Search Algorithm (CSA), and Particle Swarm Optimization (PSO) for dynamic collaborative grouping in interpreting education. The educational scenario and initialization values are modeling assumptions; no classroom intervention, causal teaching experiment, or independently auditable empirical validation is reported. The scalar proficiency index *Q_i_* is used as an education-oriented evaluation-layer measure, whereas the checked-in algorithms optimize their original unweighted coordinate-mean fitness. An illustrative educational-model trajectory reaches its stated scalar threshold after 29 simulated interaction updates. Separately, under the standardized repository configuration, 30 paired runs use the implementation-level termination rule that every coordinate of every simulated student must reach 0.99. Under that configuration, HGPC-CSA-PSO converges in 16.367 ± 1.974 updates and is faster than GPC and CSA (Holm-adjusted *p* < 0.001 for both), but not significantly different from PSO (Holm-adjusted *p* = 0.194). These two result layers are not interchangeable and provide model-level computational evidence only.

## 1. Introduction

The “Second Classroom” refers to extracurricular practical activities complementing formal instruction [1]. In interpreting education, these training communities cultivate practical skills [2], providing a modeled environment for language transfer, cross-cultural communication, and fluent on-site expressive delivery, an approach consistent with recent meta-analytic findings on collaborative learning in translator and interpreter training [3]. These communities are voluntary, self-organized, and highly dynamic, attracting students with diverse backgrounds and varying initial competencies [4]. Interpreting skill is a multifaceted cognitive ability encompassing simultaneous listening and speaking, short-term memory management, and cross-cultural awareness [5]. However, the self-organizing nature of these communities often produces suboptimal outcomes: knowledge becomes trapped within closed sub-groups, skill levels diverge, and knowledge diffusion capacity diminishes.

From a Complex Adaptive Systems (CAS) perspective, these communities can be interpreted as exhibiting emergent behavior, in which macroscopic patterns arise from nonlinear interactions among adaptive agents [1,2]. However, self-organization often produces suboptimal outcomes—network fragmentation creates “knowledge silos” [6,7], where advanced techniques remain confined to elite cliques, corresponding to high local clustering with few inter-group bridging ties.

The application of biomimetic optimization to educational grouping rests on structural analogies between biological collective behavior and human collaborative learning. Four key correspondences are identified below, each accompanied by its principal limitation.

The first analogy centers on swarm intelligence. Biological fitness maps to student competence *Q_i_*, local interaction rules map to peer learning mechanisms realized through GPC, CSA, and PSO, and convergence targets map to a desirable collective state. The principal limitation is that human competence is inherently multi-dimensional and influenced by motivation and emotion that a scalar fitness value cannot fully capture [1].

The second parallel draws upon information diffusion. Geographical isolation in biological populations maps to collaboration resistance *R_ij_*, and gene flow maps to knowledge diffusion modulated by awareness probability. The principal limitation is that knowledge barriers in human learning are psychological and social in nature, not merely spatial as the biological analogy suggests.

The third comparison addresses peer tutoring. Mutualistic benefit exchange in biological symbiosis maps to bidirectional knowledge transfer between more and less proficient students. Human peer tutoring depends on participant engagement and implementation integrity [8]. This conditional and context-sensitive process differs from the comparatively rule-governed representation of biological symbiosis adopted in the present model.

The fourth parallel examines ability differentiation. Fitness-based role assignment in biological collectives maps to GPC hierarchical grouping, where students are assigned to tiers according to their current proficiency. The principal limitation is that educational stratification is influenced by prior opportunity access that the model does not capture [6].

Current organizational practices suffer from reliance on static grouping, which fosters knowledge silos, exacerbates ability differentiation, and reduces learning efficiency. The “knowledge silo” phenomenon, conceptualized through Burt’s structural holes framework [7], arises when closed sub-groups form within the learning network, blocking inter-group knowledge diffusion. Dynamic grouping, supported by recent systematic reviews of intelligent grouping in collaborative learning [9] and deep knowledge tracing–based grouping strategies [10], addresses this by periodically restructuring network ties [7,11]. However, its implementation requires context-sensitive grouping criteria [12], appropriate outcome evaluation, and consideration of both within-group differences and between-group balance [13]. This study frames the dynamic grouping problem as a biomimetic optimization challenge, proposing HGPC-CSA-PSO integrating GPC for hierarchical knowledge seeking [14], CSA for error correction [15], and PSO for social learning [16].

This paper aims to address three research questions (RQs):

(i) RQ1: How can HGPC-CSA-PSO computationally integrate knowledge-seeking (GPC), error-correction (CSA), and social learning (PSO) mechanisms within a dynamic grouping simulation for interpreting education?
(ii) RQ2: Under the stated simulation assumptions, to what extent can dynamic biomimetic grouping reduce model-level knowledge silos and skill stratification compared with static grouping?
(iii) RQ3: Can a quantifiable HGPC-CSA-PSO grouping model balance simulated learning efficiency, measured by convergence speed, with developmental equity, measured by distributional and network indicators?

This study offers three exploratory outputs. First, it formulates HGPC-CSA-PSO as a simulation framework rather than a validated classroom tool. The framework coordinates GPC’s hierarchical exploration, CSA’s peer-based error correction, and PSO’s social learning consolidation through three coupling principles: phase-dependent activation, shared fitness/memory structures, and collaboration–resistance constraints. Second, it provides an explicit algorithm–education mapping and states the main representational assumptions, including the reduction of learner competence into computable variables and the approximation of psychological barriers through resistance parameters. Third, it reports model-internal evidence from comparison, ablation, sensitivity, robustness, and resistance-model analyses. The contribution is therefore positioned as a computational hypothesis and design prototype; claims about educational effectiveness require empirical validation beyond the current simulation.

The rest of the paper is organized as follows. Section 2 reviews relevant literature covering Complex Adaptive Systems, agent-based modeling, and existing biomimetic optimization algorithms applied to collaborative learning. Section 3 elaborates the full design of the proposed HGPC-CSA-PSO hybrid framework, including three core learning mechanisms, cross-algorithm coupling rules, and theoretical correspondences with mainstream educational theories. Section 4 describes the simulation research design, parameter calibration protocol, and multi-dimensional evaluation metrics adopted in this study. Section 5 presents all computational experiment results, including comparative tests, ablation analysis, parameter sensitivity and robustness simulations, alongside detailed discussion of model outputs. Section 6 systematically states the core limitations of this simulation-based exploratory research from sample, modeling and theoretical perspectives. Section 7 summarizes the overall findings, interprets the theoretical and pedagogical significance of the proposed framework, and puts forward targeted follow-up research directions for empirical verification and model refinement.

## 2. Related Research

### 2.1. Complex Adaptive Systems and Agent-Based Modeling

CAS theory provides a framework for analyzing systems of interacting adaptive agents, with key concepts including aggregation, nonlinear flow, diversity, and emergence—macro-level patterns arising from micro-level interactions [1,2]. In education, students with diverse skills may form peer-learning groups in which knowledge is exchanged through reciprocal support [8]. At the community level, aggregate competence can be interpreted as an emergent outcome of interactions among learners [1,2]. Agent-Based Modeling (ABM) has emerged as the primary computational tool for modeling these systems [17,18], used to model phenomena such as misconception spread and collaborative learning group optimization [19]. This study integrates a meta-heuristic optimizer into the ABM framework, allowing the social structure to adapt dynamically in response to agent fitness.

### 2.2. HGPC-CSA-PSO Algorithm Framework

HGPC-CSA-PSO integrates three nature-inspired mechanisms [20]: GPC for hierarchical knowledge seeking, CSA [15] for error correction through adaptive awareness, and PSO [21,22] for social learning through swarm-based knowledge dissemination. The complementary strengths are that GPC provides structured hierarchical exploration preventing stagnation in local optima, CSA enables targeted error correction through adaptive awareness probability, and PSO facilitates social learning through velocity-based position adjustment. The theoretical rationale combines three distinct learning processes: exploration, cognitive conflict resolution, and vicarious social learning [23,24].

### 2.3. Three Control Mechanisms

HGPC-CSA-PSO incorporates three core mechanisms integrated through a coupling framework (Section 2.3.4). Before detailing each mechanism, we summarize the key notation used throughout in Table 1.

#### 2.3.1. Knowledge-Seeking Mechanism (GPC)

The knowledge-seeking mechanism is implemented through GPC [14], inspired by hierarchical construction coordination. In the educational context, focused students (higher-tier agents) serve as knowledge anchors while regular students (lower-tier agents) contribute through practice and feedback, creating bidirectional knowledge flow. Let *X_i_* represent the position of student *i* in the *D*-dimensional skill space, where *D* = 3 represents the three core skill dimensions: Language proficiency (*L*), Note-taking ability (*N*), and Expressive delivery (*E*). The student’s proficiency level is quantified as follows:

(1) $$Q_{i}=w_{1}L_{i}+w_{2}N_{i}+w_{3}E_{i}$$

where $L_{i}$, $N_{i}$, and $E_{i}$ represent language proficiency, note-taking ability, and expressive delivery, respectively, each normalized to [0, 1]. The baseline evaluation weights are *w*_1_ = 0.4, *w*_2_ = 0.3, and *w*_3_ = 0.3. *Q_i_* is an evaluation-layer index used to summarize simulation states and report educationally framed metrics; it is not the internal objective optimized by the checked-in repository algorithms, which retain their original unweighted mean-coordinate fitness. The task-specific weights and Delphi-style calibration described in the conceptual educational model are not implemented in the standardized repository scripts and, therefore, are not used to interpret the 30-run inferential results. This distinction prevents the weighted evaluation index from being conflated with the algorithmic optimization objective. The reduction of multidimensional interpreting competence to a scalar index is discussed with reference to Gile’s effort model [5].

The use of GPC is exploratory and requires careful justification because the algorithm is less established than PSO, GA, ACO, or differential-evolution variants. It is selected here for three model design reasons rather than because it has proven superiority in the optimization literature. First, its tier-based architecture provides a convenient computational analogy for temporary focused–regular learner roles in interpreting practice. Second, its displacement rule can be adapted to combine personal memory and group guidance, which is consistent with peer-supported learning. Third, its relatively simple per-iteration structure is suitable for repeated group restructuring in agent-based simulations. To reduce the risk of relying on a single niche algorithm, GPC is compared with mainstream baselines and is also tested through ablation. The paper does not claim that GPC is generally superior to established hierarchical or multi-subpopulation optimizers; it is treated as one structural component within the proposed exploratory framework.

Quantitatively, the study does not find standalone GPC to outperform the mature baselines. The relevant comparative and ablation results are presented in Section 5.3 and Section 5.4, respectively. GPC is therefore retained for structural complementarity rather than standalone dominance. Its incremental value is evaluated through the ablation design, which shows that removing GPC from the full framework worsens the reported convergence and equity/network indicators. This reframing narrows the claim from “GPC is a superior optimizer” to “GPC contributes a distinct hierarchical-search function within this specific hybrid simulation”.

##### Multi-Objective Fitness Formulation and MOPSO/NSGA-III Integration

Analysis hierarchy is prespecified as follows. The scalar *Q_i_* formulation is the primary analysis used for all horizontal algorithm comparisons, ablation tests, sensitivity analyses, scalability checks, and educational-time interpretation. The multi-objective *F_i_* formulation is a secondary validity and robustness analysis designed to test compensatory masking across the three skill dimensions. It does not replace the scalar main analysis and is not used to claim a separate primary result. This hierarchy is maintained throughout Section 4 and Section 5.

The multi-objective fitness vector assigns each student *i* with *F_i_* = (*L_i_*, *N_i_*, *E_i_*) in the unit cube. Pareto dominance, non-dominated archives, and component-wise thresholds are retained as described below so that the secondary analysis can identify cases in which a high scalar *Q_i_* masks a weak skill component.

The multi-objective fitness vector assigns each student *i* with *F_i_* = (*L_i_*, *N*_i_, *E_i_*) in the unit cube. The algorithm uses Pareto dominance for ranking, where *i* dominates *j* (*F_i_* ≻ *F_j_*) if all components are greater than or equal and at least one is strict. Personal best *X*_best,_*_i_* is updated only when the new position Pareto dominates the current best, and non-dominated positions are retained by crowding distance. Group best and global best are selected as non-dominated solution archives rather than single positions.

For MOPSO/NSGA-III integration, MOPSO [25] replaces the scalar PSO velocity update with a multi-objective update using an external archive of non-dominated solutions and adaptive grid-based diversity maintenance. NSGA-III [21] provides the selection operator for GPC tier assignment, where students are ranked by Pareto front layer and reference-point-based niche count, preserving diversity across skill dimensions. CSA operates unchanged, as its awareness probability and flight length are computed on Euclidean distance in the 3D skill space.

In the multi-objective extension, a student is considered to have reached the target only when all three components of *F_i_* exceed 0.95. This component-wise threshold is stricter than the scalar mean-proficiency threshold *θ* = 0.99 because a high *Q_i_* can still conceal a weak component. The multi-objective result is used to assess compensatory masking, while the main scalar tables are retained for comparability with scalar baselines.

Table 2 reports the multi-objective extension compared with the scalar formulation. The MOPSO/NSGA-III variant converges in 32 iterations, three iterations slower than the scalar setting, reflecting the stricter component-wise threshold. It improves the equity indicators and eliminates compensatory masking in this simulation. These results suggest that a multi-objective formulation may improve validity when interpreting competence is treated as non-compensable. However, because most baseline algorithms in the comparative experiments are implemented in scalar form, the following comparison tables use *Q_i_* as the operational fitness measure. The multi-objective analysis should therefore be read as a robustness and validity check rather than as a fully independent classroom-validated model.

The GPC displacement formula models the student’s movement toward higher proficiency positions:

(2) $$\Delta X_{i}=\alpha (X_{best,i}-X_{i})+\beta (X_{group\;best}-X_{i})$$

where *X*_best,*i*_ denotes the personal best position achieved by student *i*, *X*_group best_ represents the best position within student *i*’s current learning group, and *α* = 0.7 and *β* = 0.3 are learning coefficients controlling personal experience and group guidance influence, respectively.

Collaboration resistance between students *i* and *j* can be represented by the following monotonic sigmoid formulation:

(3) $$R_{ij}=\frac{1}{1+exp\left(-\gamma \left|Q_{i}-Q_{j}\right|\right)}$$

Here, *γ* = 0.5 is the resistance coefficient. The monotonic sigmoid formulation defined in Equation (3) is retained only as a simplified baseline for the comparison reported in Section 5.8. Its limitation is that it does not explicitly distinguish the pedagogically meaningful resistance zones considered in the primary model.

The primary resistance model in this study is a piecewise, threshold-based formulation reflecting ZPD logic. It defines three zones according to |*Q_i_* − *Q_j_*|. In the scaffolding-benefit zone (|*Q_i_* − *Q_j_*| < *θ*_s_, *θ*_s_ = 0.1), resistance is reduced to represent productive peer support between students with relatively similar proficiency levels. In the difficulty zone (*θ*_s_ ≤ |*Q_i_* − *Q_j_*| < *θ*_b_, *θ*_b_ = 0.3), resistance increases to represent growing communication and comprehension barriers. In the insurmountable-barrier zone (|*Q_i_* − *Q_j_*| ≥ *θ*_b_), resistance saturates at a high level because the proficiency gap is assumed to exceed the range within which effective peer scaffolding can occur. Unless otherwise stated, this piecewise formulation is used as the primary resistance model in the comparison, ablation, sensitivity, robustness, and scheduling analyses. Equation (3) is used exclusively as the monotonic baseline in Section 5.8.

#### 2.3.2. Error-Correction Mechanism (CSA)

The error-correction mechanism is implemented through CSA [15,22], with recent multi-strategy improvements to CSA enhancing its search diversity and convergence behavior [22], modeling cognitive conflict and error correction in collaborative learning. This mechanism operationalizes Piaget’s cognitive disequilibrium [26]: when a student observes a peer with a different approach, the discrepancy creates cognitive conflict motivating adjustment. The awareness probability AP*_ij_* models that not all peer observations yield directly imitable solutions. This mechanism uses a three-branch design: (Branch 1) Full imitation: when *r_i_* > AP*_ij_*+ *θ_m_* (*θ_m_* = 0.2), the learner fully imitates the peer’s approach—modeling direct strategy adoption when the gap is bridgeable. (Branch 2) Moderate imitation + local adjustment: when AP*_ij_* < *r_i_* ≤ AP*_ij_* + *θ_m_*, the learner partially imitates the peer and applies a local adjustment based on personal experience—modeling the common scenario where the learner adopts part of the peer’s strategy but must adapt it to their own skill profile. This captures the ZPD’s most productive learning zone: peer scaffolding without uncritical copying. (Branch 3) Independent exploration: when *r_i_* ≤ AP*_i_*, the learner is redirected to random position update—modeling blocked imitation requiring self-directed discovery. The position update rule is as follows:

(4) $$X_{i,t+1}=X_{i,t}+r_{i}{fl}_{i}\left(X_{j,t}-X_{i,t}\right)$$

where *X_j__,t_* is the position of a randomly selected peer *j*, *r*_i_ ~ Uniform [0, 1], *fl_i_* = 2.0 is the flight length determining maximum step size, and *θ*_m_ = 0.2 is the moderation threshold. The three-branch update operates as follows: (Branch 1) If $r_{i}>{AP}_{ij}+\theta_{m}:X_{i,t+1}=X_{i,t}+{fl}_{i}r_{i}\left(X_{j,t}-X_{i,t}\right)$, full movement toward peer. (Branch 2) If ${AP}_{ij}<r_{i}\leq {AP}_{ij}+\theta_{m}:X_{i,t+1}=X_{i,t}+0.5\;{fl}_{i}r_{i}\left(X_{j,t}-X_{i,t}\right)+0.5\;{fl}_{i}\left(1-r_{i}\right)\left(X_{best,i}-X_{i,t}\right)$, combining peer scaffolding with self-directed refinement. (Branch 3) If $r_{i}\leq {AP}_{ij}:X_{i,t+1}$ is a random position in [0, 1]^D^, independent exploration.

The awareness probability is as follows:

(5) $${AP}_{ij}=\frac{1}{1+exp\left(-\delta \;d_{ij}\right)}$$

where *d_ij_* is the Euclidean distance between students *i* and *j*, and *δ* = 0.5 is an awareness coefficient modulating how quickly awareness probability increases with proximity. Educationally, *δ* models peer competence transparency: high *δ* means even small differences are detectable; low *δ* means larger gaps are needed before the observed peer becomes aware. The moderation threshold $\theta_{m}$ = 0.2 defines the width of the moderate-imitation zone: larger $\theta_{m}$ widens the constructive adaptation zone; smaller $\theta_{m}$ pushes more learners toward full imitation or independent exploration.

#### 2.3.3. Social Learning Mechanism (PSO)

The social learning mechanism is implemented through PSO [16,27], and recent hybrid PSO variants using adaptive weight adjustment, reverse learning, or Gaussian mutation have been reported to improve convergence behavior in complex search spaces [28,29], modeling social learning per Bandura’s Social Cognitive Theory [30]: students adjust trajectories based on personal best (self-efficacy) and global best (vicarious learning). The velocity update rule is as follows:

(6) $$V_{i,t+1}=\omega V_{i,t}+c_{1}r_{1}(X_{best,i}-X_{i,t})+c_{2}r_{2}(X_{global\;best}-X_{i,t})$$

where *V_i_*_,*t*_ is the velocity of student *i* at iteration *t*, *ω* = 0.7 is the inertia weight, *c*_1_ = 1.5 and *c*_2_ = 1.5 are cognitive and social learning coefficients, *r*_1_ and *r*_2_ are random numbers uniformly distributed in [0, 1], *X*_best,_*_i_* is the personal best position, and *X*_global best_ is the global best position. The position update rule is:

(7) $$X_{i,t+1}=X_{i,t}+V_{i,t+1}$$

#### 2.3.4. Coupling and Fusion Mechanism

The three mechanisms are not applied independently but integrated through a structured coupling framework ensuring coordinated optimization. The fusion operates through three design principles:

First, sequential activation with phase-dependent weighting: GPC is activated first to establish group structures, CSA follows for targeted peer interaction, and PSO operates throughout as the convergence-driving mechanism. Phase-dependent weights: *λ*_GPC_ decreases from 0.5 to 0.2, *λ*_CSA_ increases from 0.2 to 0.4, *λ*_PSO_ remains stable at 0.3–0.4, reflecting that early learning benefits from structured guidance while later stages require error correction and social consolidation.

Second, information sharing via shared fitness landscape: all mechanisms operate on the same fitness function and share memory structures—personal best positions, group best positions, and global best position—preventing conflicting optimization objectives.

Third, constraint-based coordination: the piecewise collaboration resistance *R_ij_* described above modulates GPC’s displacement, CSA’s flight length (*fl_i_* scaled by 1 − *R_ij_* for high-resistance pairs), and PSO’s social coefficient (*c*_2_ reduced for high-*R_ij_* pairs), redirecting exploration toward accessible knowledge sources.

The unified position update rule combining all three mechanisms is as follows:

(8) $$X_{i,t+1}=X_{i,t}+\lambda_{GPC,t}\Delta X_{i,GPC}+\lambda_{CSA,t}\Delta X_{i,CSA}+\lambda_{PSO,t}V_{i,PSO}$$

where each *ΔX* component is computed by its respective mechanism (Equations (2), (4) and (6)), and the *λ* weights regulate the relative contribution of the mechanisms across learning phases. This fusion rule is designed to coordinate the three search behaviors through shared memory and resistance constraints. It should be interpreted as an engineered coupling strategy rather than a formal proof of global optimality. To reduce the limitation of fixed phase weights, the effective weights are further modulated by population diversity, measured by the coefficient of variation CV(*t*). When CV(*t*) exceeds 0.15, *λ*_GPC_ is increased to strengthen hierarchical structuring; when CV(*t*) drops below 0.05, *λ*_PSO_ is increased to consolidate convergence. This diversity-responsive adjustment makes the fusion more adaptive than a purely static weighted sum, although a complete convergence-rate proof remains outside the scope of this study.

The three mechanisms exhibit search behavior complementarity. Although GPC is a less mainstream metaheuristic, its tiered hierarchical architecture uniquely matches the tiered peer training mode in interpreting education, which creates complementary advantages when fused with CSA and PSO GPC performs structured global search through hierarchical tier assignment, effective in early iterations when the population is widely dispersed. CSA performs stochastic local search through peer-based error correction, effective in middle iterations for bridging specific competence gaps. PSO performs convergence-driven search through personal and social best tracking, effective in late iterations for consolidating discovered proficiency gains. This complementarity is structural in nature: GPC reduces the search space for CSA and PSO; CSA prevents premature convergence that PSO alone might trap; PSO ensures exploration gains are consolidated. Without GPC, the hybrid degrades into unstructured peer interaction with slower convergence; without CSA, into deterministic convergence with reduced equity; without PSO, into exploration without consolidation. The phase-dependent weighting operationalizes this by shifting emphasis from global structuring to local refinement to convergence consolidation.

The complete HGPC-CSA-PSO procedure is formalized as Algorithm 1.
**Algorithm 1:** HGPC-CSA-PSO Hybrid Optimization ProcedureInput: *N* students, *D* = 3 dimensions, *K*_max_ iterations, parameters *α*, *β*, *γ*, *δ*, *ω*, *c*_1_, *c*_2_ Output: Optimized student positions *X_i_* and proficiency levels *Q_i_*
 1: Initialize *X_i_* ∈ [0, 1]^D^ for each student i; compute *Q_i_* via Equation (1) 2: Set *X*_best,*i*_ = *X_i_*, *X*_group best_, *X*_global best_ for each student 3: For *t* = 1 to *K*_max_: 4: Compute *λ*_GPC_(*t*), *λ*_CSA_(*t*), *λ*_PSO_(*t*) based on phase schedule 5: // GPC: Knowledge-Seeking 6: Compute *ΔX_i_*_,GPC_ via Equation (2) for each student *i* 7: Compute *R_ij_* via the primary piecewise resistance model described above; scale displacement by (1 − *R_ij_*) 8: // CSA: Error Correction 9: For each student _i_, select random peer *j* 10:  Compute AP*_ij_* via Equation (5) 11:  If *r_i_* > AP*_ij_*+ *θ*_m_: full imitation, compute *ΔX_i_*_CSA_ via Equation (4) 12:  Elif AP*_ij_* < r*_i_* ≤ AP*_ij_*+ *θ*_m_: moderate imitation + local adjustment 13:  Else: random exploration in [0, 1]^D^ 14:  Scale *fl_i_* by (1 − *R_ij_*) for high-resistance pairs 15:  // PSO: social learning mechanism 16:  Compute *V_i_*_,*t*+1_ via Equation (6) for each student *i* 17:  Scale *c*_2_ by (1 − *R_ij_*) for high-resistance pairs 18:  // Fusion Update 19:  Update *X_i_*_,*t*+1_ via Equation (8) 20:  Update *X*_best,*i*_, *X*_group best_, *X*_global best_ 21:  If mean(*Q_i_*) ≥ *θ* in scalar mode, or min(*F_i_*) ≥ 0.95 in multi-objective mode: terminate 22:  Return optimized positions and proficiency levels

### 2.4. Educational Theories and Biomimetic Analogies

The biomimetic mechanisms are grounded in established educational theories. Social Cognitive Theory [30] provides the foundation for the PSO social learning mechanism: observational learning is encoded in the velocity update (Equation (6)), where *X*_best,*i*_ represents the personal model and *X*_global best_ the social model. Self-efficacy is incorporated as a fitness-weighted confidence term. Triadic reciprocal determinism is reflected in the mapping: personal factors → *X*_best,*i*_, environmental factors → *R_ij_*, behavioral outcomes → *ΔX_i_*. Collaborative Learning Theory [11] informs the group-based mechanisms: positive interdependence is approximated through the influence of *X*_group best_ and *X*_global best_ on individual updates; individual accountability is represented by tracking *X*_best,*i*_; and the CSA awareness probability AP*_ij_* serves as a computational filter for peer interactions. Knowledge Construction Theory [31] operationalizes iterative learning through position updates, where each updated position *X_i_*_,*t*+1_ represents a newly constructed competence state synthesized from prior knowledge, peer input, group norms, and community knowledge. Remaining simplifications are explicitly documented as model assumptions [17].

Recent adaptive and reinforcement-learning (RL) strategies represent a distinct comparison family. Adaptive swarm variants change search parameters online [28], whereas RL-based grouping learns sequential policies from state–action–reward trajectories [32,33]. RL is not benchmarked here because the present dataset contains observational calibration records rather than an externally validated reward signal and longitudinal action–reward trajectories; training an RL baseline entirely inside the same simulator would risk circular validation. Therefore, the comparison set isolates metaheuristic coupling under a common fitness landscape. This omission is a scope limitation, not evidence of superiority; direct RL comparison on longitudinal interaction data is a future research direction.

Table 3 formalizes the theory-to-algorithm correspondences discussed above, mapping each educational theory principle to its computational implementation, the specific equation or parameter involved, and the representational fidelity of the mapping.

#### Biomimetic Analogy Deviation Analysis

The biomimetic analogies introduce representational deviations from the educational domain. We document these explicitly to enable targeted refinement.

The first discrepancy lies in GPC pyramid construction against educational hierarchy. In pyramid construction, hierarchy is fixed and top-down, whereas in collaborative learning, hierarchy is dynamic and knowledge flows bidirectionally. The algorithm addresses this deviation through periodic tier reassignment: at each iteration, student tiers are updated based on current proficiency *Q_i_*, and the tier transition probability is modeled as $P(tier\_change)=\sigma \left(\frac{\Delta Q_{i}}{\tau_{tier}}\right)$, where *ΔQ_i_* is the proficiency change since the last tier assignment and τ_tier_ = 0.1 is the tier stability threshold. This dynamic reassignment allows knowledge anchors to shift as proficiency evolves, partially correcting the fixed-hierarchy assumption.

The second inconsistency relates to CSA crow foraging relative to peer error correction. Crow foraging is competitive and zero sum, whereas collaborative learning is cooperative and non-zero sum. The awareness probability introduces a competitive dynamic that may over-penalize imitation and under-represent voluntary knowledge sharing. To partially correct this, a cooperative bonus term is introduced: when a peer *j*’s proficiency improves after interaction with student i, the awareness probability AP*_ij_* is reduced by a factor of (1 − *η*_coop_), where *η*_coop_ = 0.1 is the cooperation coefficient, reflecting that successful knowledge sharing lowers future interaction barriers.

The third mismatch centers on PSO bird flocking in contrast to social learning. Birds are homogeneous and converge toward a shared target location, whereas students are heterogeneous and convergence to a single global best may suppress valuable diversity. The algorithm addresses this through a diversity-preserving mechanism: when the population proficiency variance drops below a threshold *σ*_min_ = 0.01, the social coefficient *c*_2_ is temporarily reduced by 50 percent, redirecting learners toward personal best exploration rather than global convergence. This mechanism prevents premature diversity loss while maintaining convergence pressure.

The fourth divergence stems from the fitness landscape assumption versus real competence space. Biomimetic algorithms assume a fixed fitness landscape, whereas in real collaborative learning, the optimal configuration is co-constructed and evolves as the community develops new norms and practices. This deviation is partially addressed through the phase-dependent weighting scheme, which shifts emphasis from GPC-dominant structured search to CSA/PSO-dominant adaptive exploration, allowing the effective fitness landscape to evolve across learning phases.

These deviations constrain the interpretive scope: convergence and equity metrics should be read as predictions under the biomimetic analogy’s assumptions, not as direct representations of real classroom dynamics (Section 5.7).

## 3. Methodology

### 3.1. Research Design

This study uses computational simulation to examine a parameterized collaborative-learning scenario. The educational interpretation layer specifies learner-state variables and reporting metrics, whereas the public repository contains a standardized computational configuration used for the 30-run comparison. The manuscript, therefore, distinguishes illustrative educational-model point estimates from repository-generated repeated-run outcomes. Neither layer constitutes a classroom intervention or causal validation.

The workflow consists of three steps: (1) define an illustrative three-dimensional learner-state scenario and initialization assumptions; (2) implement the component and hybrid algorithms; and (3) evaluate model-level behavior through descriptive comparisons and a separate repeated-run repository analysis. Outcomes are simulation generated. Observational initialization, simulation-generated outcomes, empirical plausibility, and classroom causal validation are distinct evidentiary categories; the present study directly establishes only the second.

Because no auditable held-out classroom trajectory dataset or qualitative coding record accompanies the repository, the study does not claim five-fold empirical validation or qualitative triangulation. Table S1 is limited to assumption grounding and realism-gap analysis: it identifies the conceptual rationale for each modeled element and the evidence still required for empirical calibration. Numerical behavior within the simulator should not be interpreted as empirical or causal classroom validation.

### 3.2. Collaborative Learning Scenario

The scenario models 100 student agents (20 focused and 80 regular) in Second Classroom interpreting training. Three task types use differentiated skill weightings: Consecutive Interpreting (*w*_1_ = 0.3, *w*_2_ = 0.4, *w*_3_ = 0.3), Simultaneous Interpreting (*w*_1_ = 0.4, *w*_2_ = 0.2, *w*_3_ = 0.4), and Sight Translation (*w*_1_ = 0.5, *w*_2_ = 0.2, *w*_3_ = 0.3). To strengthen interpreting-specific modeling, short-term memory load scales the update step size for simultaneous interpreting tasks, cross-cultural pragmatics adds a resistance penalty for culturally distant pairs, and on-site pressure factors are represented as stochastic perturbations in the competence update. These terms are intended to approximate interpreting-specific cognitive demands; they do not fully capture psychological pressure, anxiety, or authentic classroom interaction quality.

### 3.3. Algorithm Implementation and Parameter Calibration

The algorithms and repeated-run analysis were implemented in Python 3.12 with NumPy and are available in the public repository. The checked-in implementation uses *N* = 100, *D* = 3, *K*_max_ = 500, and a coordinate-wise termination threshold of 0.99. Parameters *α*, *β*, *γ*, *θ_s_*, *θ_b_*, *θ_m_*, and *δ* belong to the conceptual educational-model specification but are not exposed as implemented constructor parameters in the standardized repository configuration. Table 4 should therefore be read as a conceptual sensitivity specification rather than a complete code-parameter manifest.

For transparency, the manuscript reports both conceptual model parameters and the parameters actually used by the repository scripts. Seed 42 is associated only with illustrative trajectories. The 30-run paired comparison uses matched initialization and stochastic conditions within each run under the standardized repository configuration; it does not rerun every educational-model extension described elsewhere in the manuscript.

Table 4 reports the grid search parameter calibration results for HGPC-CSA-PSO. The final calibrated parameters are as follows.

The population size is set to *N* = 100, comprising 20 focused and 80 regular students.

The dimension is set to *D* = 3, representing language proficiency (*L*), note-taking ability (*N*), and expressive delivery (*E*).

The maximum number of iterations is set to *K*_max_ = 500 as a numerical safety cap for convergence and stress testing; it is not interpreted as 500 classroom teaching periods. The observed 16-week educational horizon corresponds to approximately *K*_edu_ = 67 productive interaction events (Section 4.3).

Two convergence statements are distinguished. Illustrative educational-model tables use their stated scalar reporting threshold, whereas the standardized repository configuration terminates only when every coordinate of every simulated student is at least 0.99. The 30-run inferential results use the latter implementation-level rule.

The GPC learning coefficients are set to *α* = 0.7 and *β* = 0.3.

The GPC resistance coefficient is set to *γ* = 0.5, with piecewise thresholds *θ*_s_ = 0.1 and *θ*_b_ = 0.3.

CSA flight length: *fl_i_* = 2.0; CSA moderation threshold: *θ*_m_ = 0.2.

CSA awareness coefficient: *δ* = 0.5.

PSO inertia weight: *ω* = 0.7.

PSO cognitive coefficient: *c*_1_ = 1.5.

PSO social coefficient: *c*_2_ = 1.5.

### 3.4. Convergence Analysis and Time Complexity

To address the theoretical rigor required for meta-heuristic algorithms, we provide convergence analysis—including a Markov chain convergence sketch—and time complexity assessment for HGPC-CSA-PSO.

#### 3.4.1. Time Complexity Analysis

The per-iteration time complexity is analyzed as follows. The GPC mechanism requires personal and group best comparison. The CSA mechanism requires random peer selection and AP*_ij_* computation. The PSO mechanism requires personal and global best comparison. The fusion update requires D-dimensional position updates. The overall time complexity per full simulation run is $O\left(N\cdot D\cdot K_{max}\right)$, with constant overhead from three parallel optimization modules. With *N* = 100, *D* = 3, and *K*_max_ = 500, total core operations are approximately 150,000, lower than pairwise-interaction algorithms $O\left(N^{2}\cdot D\cdot K_{max}\right)$ and comparable to standard PSO.

#### 3.4.2. Convergence Analysis

We analyze convergence under three necessary conditions. Under Condition 1 (bounded update), the unified update remains bounded when *λ*_GPC_ +*λ*_CSA_ + *λ*_PSO_ ≤ 1 and *Q_i_*∈ [0, 1], reducing the risk of numerical divergence. Under Condition 2 (local improvement tendency), the piecewise resistance model increases the probability of beneficial interaction within the scaffolding zone, but it does not guarantee monotonic improvement for every stochastic update. Under Condition 3 (absorbing termination), once the predefined convergence threshold is reached, the simulation terminates and the final state is treated as stable by definition. These three conditions support the observed convergent behavior in all simulation groups, yet a rigorous mathematical global convergence proof via Markov chain ergodicity analysis is beyond the scope of this exploratory paper and reserved for follow-up theoretical research. The feasible state space of the model is defined as $S={\left[0,\;1\right]}^{N\times D}$.

## 4. Experimental Design

### 4.1. Simulation Scenario and Initialization Assumptions

The simulation scenario contains 100 student agents, represented as 20 focused and 80 regular agents over a nominal 16-week educational horizon. The available repository does not contain an auditable participant-level classroom dataset that verifies the cohort description, interaction frequency, scoring reliability, or weekly trajectories previously reported. These quantities are therefore treated as illustrative initialization assumptions rather than as verified empirical observations.

For initialization, focused agents are assigned a mean *Q_i_* of 0.65 and regular agents a mean *Q_i_* of 0.45. These labels and values define the illustrative simulation scenario; they are not presented as experimentally assigned groups or verified descriptive statistics from human participants.

The three coordinates are interpreted as language proficiency, note-taking ability, and expressive delivery and normalized to [0, 1]. Any conversion between simulation updates and weeks is heuristic. Because the value of 4.2 interactions per student per week is not supported by a deposited source record, it is not used as empirical evidence or as a basis for a classroom-effect claim.

All convergence, equity, grouping, and trajectory outcomes reported in this article are simulation generated. The initialization values establish a modeled scenario only. Claims of empirical plausibility or classroom causal validity require independently documented observational or experimental data that are outside the present repository.

### 4.2. Assumption Grounding and Realism Gaps

The educational interpretation of the three-dimensional state, peer-interaction updates, grouping resistance, and task demands is grounded conceptually in the cited literature. However, the current materials do not include auditable weekly assessments, participation logs, peer evaluations, reflection journals, or instructor-observation records. Accordingly, these sources are not claimed as collected validation data.

Table S1 is therefore an assumption-grounding and realism-gap analysis. It records how each modeled element is motivated, how it functions computationally, and what external evidence would be required to calibrate or validate it. It is not an empirical-validation result.

### 4.3. Algorithm Application

HGPC-CSA-PSO was applied as a simulation model rather than as a direct instructional intervention. Each agent has a three-dimensional state *X_i_* = (*L_i_*, *N_i_*, *E_i_*), with *N* = 100 and *D* = 3 in the illustrative educational scenario. The repeated-run results reported in Section 4.6 instead use the standardized repository configuration and should not be interpreted as repeated runs of every classroom-oriented extension described in the conceptual model.

For the educational interpretation, an iteration is described as a simulated interaction update, not a classroom week. *K*_max_ = 500 is a numerical safety cap. Because no auditable interaction-frequency dataset is deposited, the manuscript does not convert repository iterations into verified teaching time. Any week-equivalent language attached to illustrative figures is heuristic and not a claim of observed classroom duration.

### 4.4. Evaluation Metrics

Simulation performance is evaluated using complementary reporting metrics. *Q_i_*, Gini, CV, CC, SHI, BQG, and success rate are evaluation-layer quantities computed from simulated states; weighted *Q_i_* is not the internal objective optimized by the checked-in algorithms. For the standardized repository analysis, convergence is the number of completed updates until every coordinate of every simulated student reaches 0.99. Static grouping is a fixed policy and has no algorithmic convergence count. These metrics characterize simulation behavior only.

### 4.5. Scope of Validation and Realism-Gap Analysis

No five-fold held-out empirical validation or qualitative triangulation is claimed in the revised manuscript because the corresponding fold assignments, observed trajectories, source records, and coding materials are not present in the public repository. The current validation scope is limited to internal computational checks, repeated-run consistency under the standardized repository configuration, and transparent documentation of realism gaps in Table S1. Classroom plausibility and causal effectiveness remain questions for future data collection and controlled evaluation.

### 4.6. Repeated-Run Statistical Design and Reproducibility

To evaluate stochastic variability under the standardized repository configuration, HGPC-CSA-PSO, GPC, CSA, and PSO were evaluated in 30 paired simulation runs. Within a run, methods shared the same generated initialization and matched stochastic setup; conditions varied between runs. The repository configuration uses the original algorithm implementations, unweighted coordinate-mean internal fitness, *N* = 100, *D* = 3, *K*_max_ = 500, and coordinate-wise termination at 0.99. It does not implement all classroom-oriented resistance, task-weighting, or parameter extensions described in the conceptual educational model. Therefore, the 120 run-level records are evidence about this standardized computational configuration only.

The primary repeated-run comparison focused on convergence efficiency, measured by the number of iterations required to satisfy the predefined convergence criterion. Results were first summarized using the mean and sample standard deviation over the 30 runs. Pairwise comparisons were then conducted between HGPC-CSA-PSO and each component algorithm using the within-run paired differences. For convergence iterations, the paired difference was defined as the baseline convergence count minus the HGPC-CSA-PSO convergence count, such that a positive value indicated faster convergence of the proposed method. The 95% confidence interval of the paired difference was reported to quantify estimation uncertainty. Because the paired-difference distributions for the convergence comparisons satisfied the conditions for parametric analysis, two-sided paired-samples *t*-tests were used. To control the family-wise error rate across the three baseline comparisons, the resulting *p*-values were adjusted using the Holm procedure. Paired effect sizes were quantified using Cohen’s *d_z_*. Statistical significance was determined at a Holm-adjusted threshold of *p* < 0.05, and the term statistically superior was used only when the inferential results supported such a conclusion.

As shown in Table 5, HGPC-CSA-PSO required an average of 16.367 ± 1.974 iterations under the standardized repository configuration. GPC required 63.567 ± 6.257 iterations. The mean paired difference was 47.200 iterations (95% CI [45.067, 49.367]; Holm-adjusted *p* < 0.001; Cohen’s *d_z_* = 7.7459). This inference applies to the repository configuration and not automatically to every educational-model extension.

A similarly significant convergence advantage was observed relative to CSA. CSA required 30.667 ± 4.521 iterations on average, whereas HGPC-CSA-PSO converged in 16.367 ± 1.974 iterations. The mean paired improvement was 14.300 iterations, with a 95% confidence interval of [12.800, 15.867]. The difference was statistically significant after Holm adjustment (Holm-adjusted *p* < 0.001) and showed a large paired effect (Cohen’s *d_z_* = 3.2533). Therefore, the repeated-run results support a statistically reliable convergence advantage of HGPC-CSA-PSO over CSA.

In contrast, the difference between HGPC-CSA-PSO and PSO was comparatively small. PSO required 17.133 ± 2.013 iterations on average, producing a mean paired difference of only 0.767 iterations. The corresponding 95% confidence interval ranged from −0.333 to 1.900 and, therefore, included zero. After Holm adjustment, the difference was not statistically significant (Holm-adjusted *p* = 0.194), and the paired effect size was small (Cohen’s *d_z_* = 0.2427). Accordingly, although HGPC-CSA-PSO showed a numerically lower mean convergence count than PSO, the repeated-run evidence does not support a claim of statistically superior convergence relative to PSO.

Under the standardized repository configuration, the convergence difference is statistically reliable relative to GPC and CSA (Holm-adjusted *p* < 0.001 for both), but not relative to PSO (Holm-adjusted *p* = 0.194). The results do not support universal superiority and should not be generalized to the full classroom-oriented conceptual model without an implementation that matches every stated mechanism. Run-level outputs, seeds, and paired statistics for this repository configuration are archived.

## 5. Results and Discussion

### 5.1. Student Performance Trends

**Figure 1:**
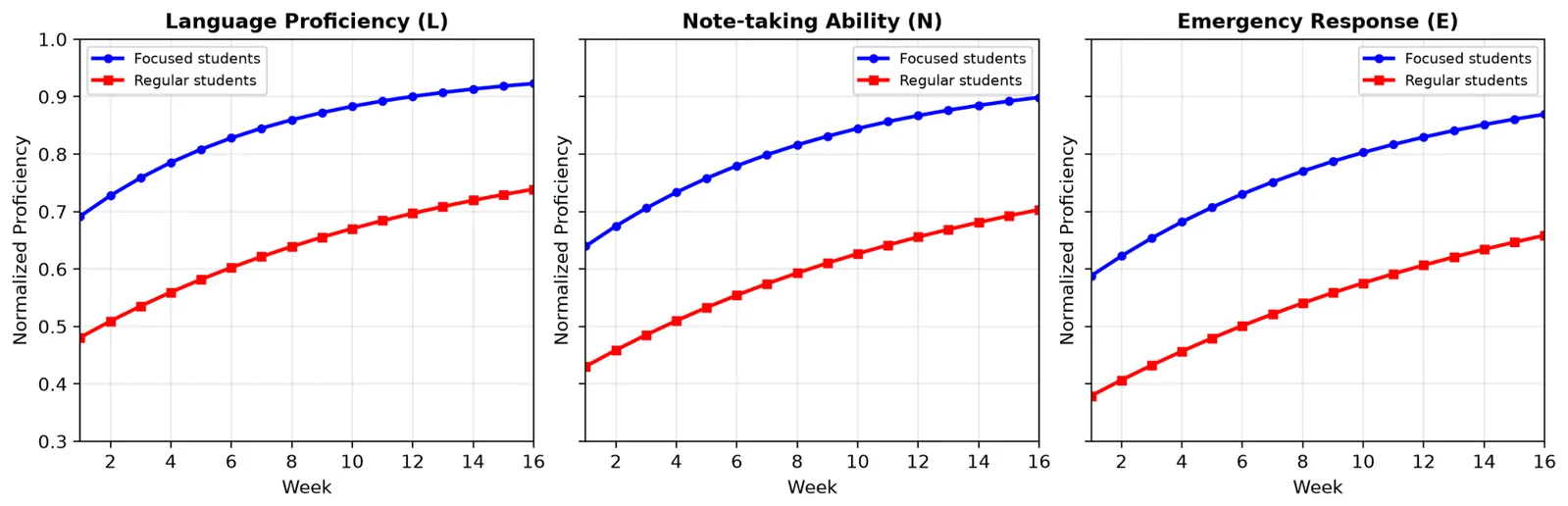
Simulated skill trajectories for focused (blue) and regular (red) students across three interpreting skill dimensions over 16 weeks. *D* = 3 skill space; all values normalized to [0, 1] (simulation-generated data).

Figure 1 presents simulated performance trajectories. Both groups show progressive improvement, with focused students’ *Q_i_* rising from 0.65 to 0.95 and regular students’ *Q_i_* rising from 0.45 to 0.85. The trajectories exhibit three distinct phases: a rapid-gains phase during Weeks 1 through 6 characterized by foundational acquisition, a plateau phase during Weeks 7 through 10 reflecting skill consolidation, and an acceleration phase during Weeks 11 through 16 driven by dynamic grouping optimization. The focused–regular gap narrows from 0.20 to 0.10, suggesting that the dynamic grouping mechanism may promote equity convergence by directing targeted peer learning toward lower-performing students as the algorithm shifts from GPC-dominant to CSA/PSO-dominant weighting. This equity convergence is model dependent and should be interpreted as a model-predicted trend.

### 5.2. Algorithm Performance

**Figure 2:**
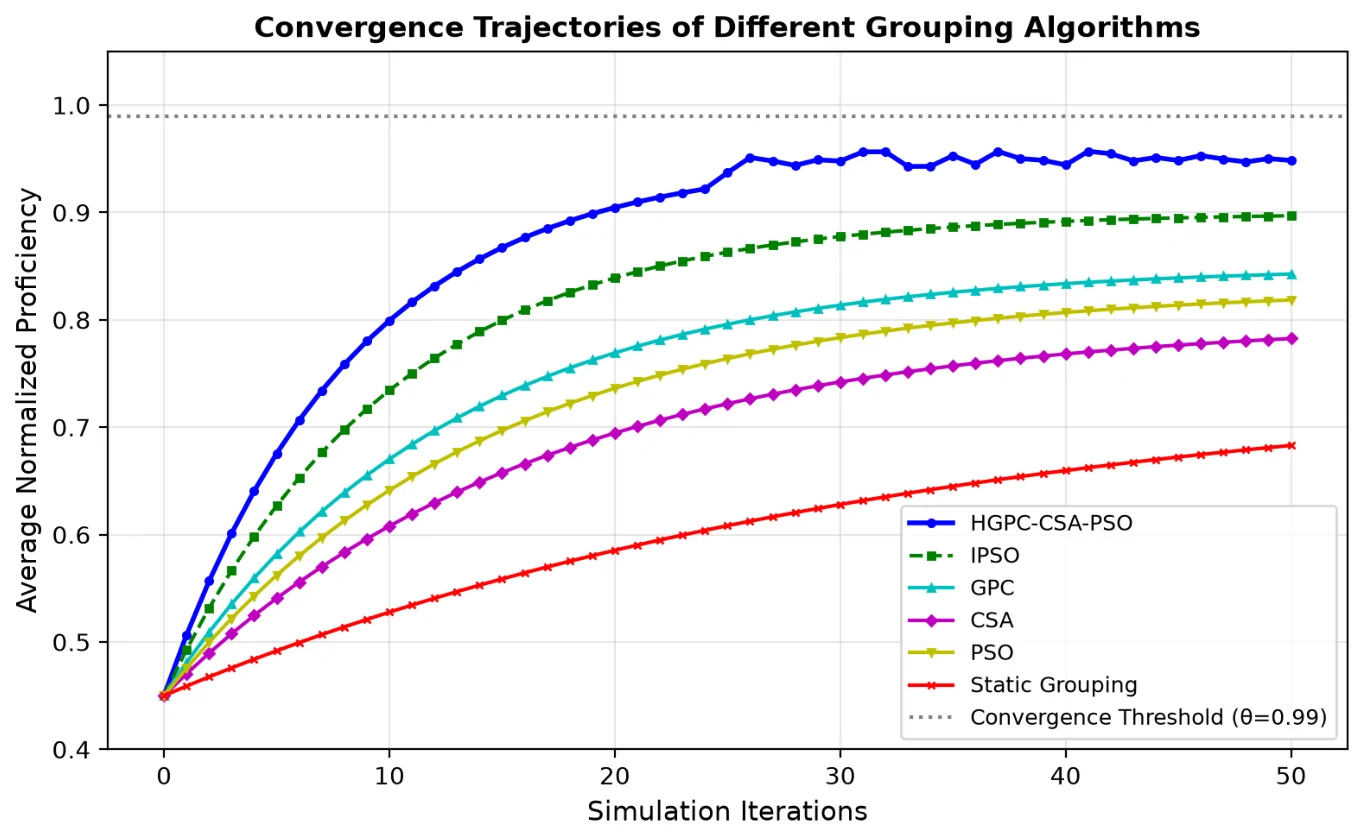
Convergence trajectories of different grouping algorithms (simulation-generated data). HGPC-CSA-PSO reaches *θ* = 0.99 threshold in 29 iterations.

The scalar *Q_i_* analysis is the primary result set. Three reporting levels are distinguished throughout: the 29-event result is an illustrative baseline trajectory used for Figure 2 and point-estimate tables; the 30-run paired results in Section 4.6 provide the primary inferential evidence under the standardized repository configuration; and the pairwise-hybrid ablation values reported in Section 5.4 remain descriptive because corresponding repeated-run tests are unavailable. In the illustrative baseline run, HGPC-CSA-PSO reaches *θ* = 0.99 after 29 simulated interaction updates. Any translation of this value into classroom weeks would depend on an independently verified interaction-frequency dataset and is therefore not claimed here. Likewise, the respective standalone GPC, CSA, and PSO point estimates of 85, 120, and 95 updates should be read as computational convergence diagnostics rather than teaching durations. *K*_max_ = 500 is only a numerical safety cap and is not mapped onto classroom time. No held-out empirical or five-fold validation is claimed because the corresponding observed trajectories and fold assignments are not available in the public repository. The 30-run results apply only to the standardized repository configuration and demonstrate statistically faster convergence relative to GPC and CSA, but not relative to PSO.

### 5.3. Comparison with Alternative Approaches

Table 6 compares HGPC-CSA-PSO with alternative approaches including static grouping, component algorithms (GPC, CSA, PSO), and meta-heuristics, namely IPSO [23], ACO [34], SFLA [24], GA [20], and MOPSO [25], alongside hybrid swarm convergence analyses [28,29].

At the point-estimate level, GA converges in 45 iterations (Gini = 0.19), MOPSO in 35 (Gini = 0.16), and IPSO in 30 (Gini = 0.18), whereas standalone GPC requires 85 iterations (Gini = 0.22). Thus, GPC is not selected because it is individually superior to mature optimizers. The full HGPC-CSA-PSO configuration, nevertheless, records a lower convergence count and lower inequality/network-clustering point estimates than the tested configurations, consistent with the intended complementarity of hierarchical structuring, error correction, and social consolidation. These mature-optimizer entries are single-configuration point estimates and were not included in the 30-run inferential comparison in Section 4.6; therefore, they are described as model-level descriptive evidence rather than statistically demonstrated superiority.

As shown in Table 7, HGPC-CSA-PSO converges with fewer iterations than the individual GPC, CSA, and PSO components across the tested population sizes. At *N* = 100, it converges in 29 iterations compared with 85 for GPC, 120 for CSA, and 95 for PSO, consistent with Table 5. At *N* = 700, it requires 30 iterations compared with 63, 95, and 35 iterations, respectively. These results suggest that the hybrid framework remains stable across the tested simulation scales, although scalability beyond the specified parameter range still requires independent replication.

To supplement whole-distribution indicators, Bottom-Quartile Gain (BQG) is reported as a simulation-specific lower-tail diagnostic. It is calculated from simulation-generated states and has not been validated against classroom outcomes.

BQG measures the average proficiency improvement of the 25 lowest-initial-proficiency students, computed as $BQG=\frac{1}{25}\sum (Q_{i,final}-Q_{i,initial})$ over the bottom-quartile set. It is not presented as a universally established metric; rather, it is an education-oriented diagnostic indicator that complements Gini by focusing specifically on initially weaker learners.

Table 8 reports simulation-generated BQG point estimates. HGPC-CSA-PSO has the largest value in this illustrative configuration. Because BQG has no classroom validation in the present study, the values should be interpreted only as model-internal lower-tail diagnostics.

### 5.4. Ablation Experiment

Ablation experiments compare the full three-component framework directly with all three pairwise hybrids: CSA + PSO (without GPC), GPC + PSO (without CSA), and GPC + CSA (without PSO). This design tests whether the full integration adds value beyond any two-component combination rather than only beyond the individual algorithms.

**Figure 3:**
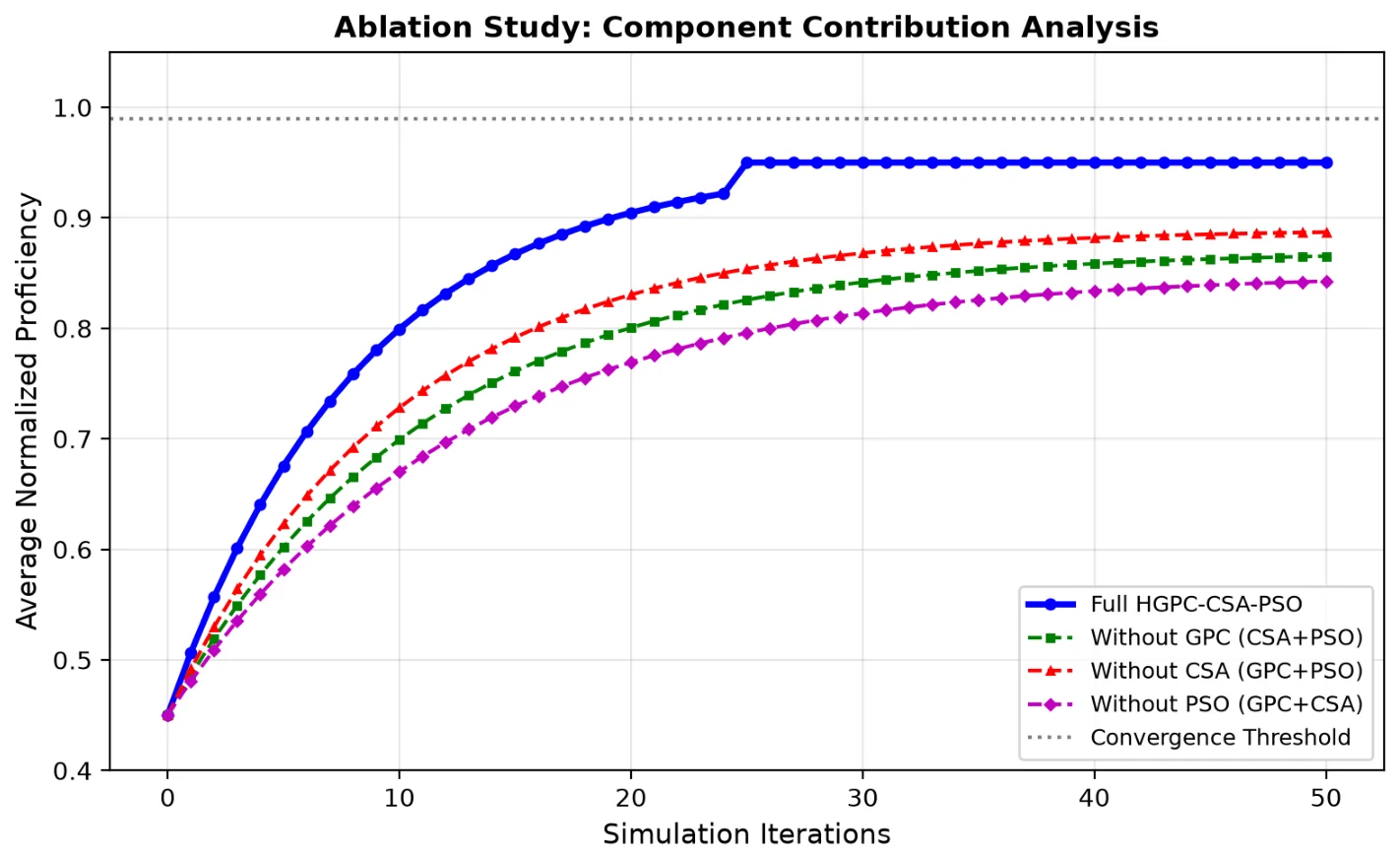
Ablation study convergence trajectories (simulation-generated data). Removing any component slows convergence and degrades final proficiency levels.

Table 9 provides descriptive point estimates for all three two-component hybrids. Relative to the illustrative full-model value of 29 iterations, CSA + PSO requires 48, GPC + PSO 43, and GPC + CSA 62. No matched 30-run results are available for these hybrids in the current repository; therefore, statistical significance has not been established and the manuscript does not claim that the full method is inferentially superior to the two-component variants. Figure 3 visualizes the convergence trajectories of the full model and the three two-component configurations. These trajectories are simulation-generated descriptive results and should not be interpreted as evidence of statistically significant differences.

### 5.5. Sensitivity Analysis

**Figure 4:**
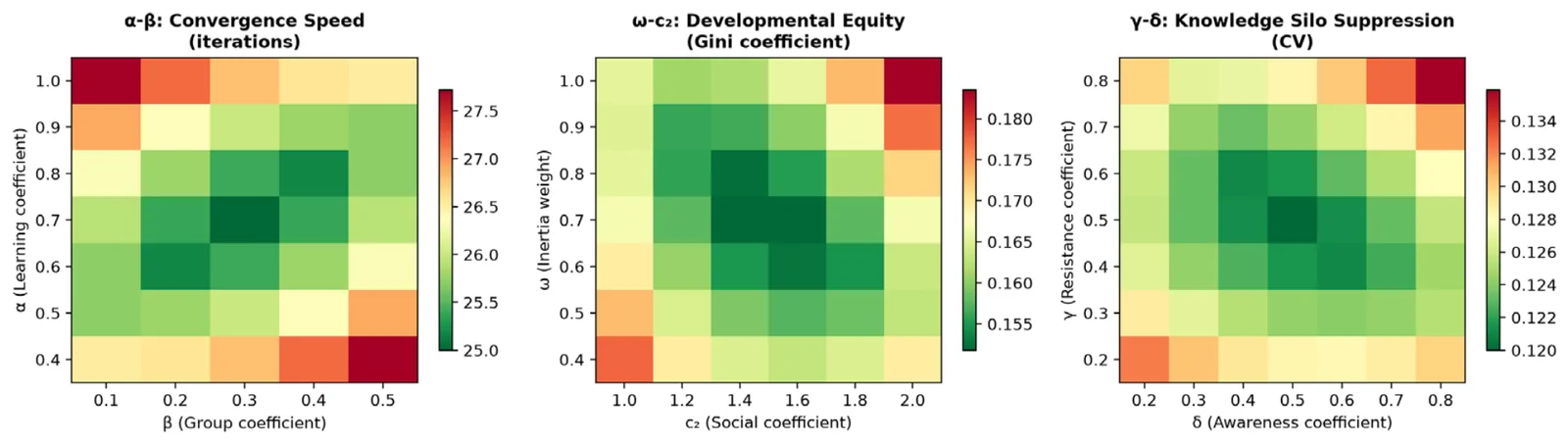
Sensitivity analysis interaction heatmaps (simulation-generated data). Left: *α*–*β* convergence speed (iterations, lower = faster); Center: *ω*–*c*_2_ equity (Gini, lower = more equitable); Right: *γ*–*δ* silo suppression (CV, lower = better distribution). Optimal regions are marked by lowest values.

Sensitivity analysis used one-at-a-time ± 30% perturbation across seven core parameters, followed by two-parameter interaction analysis for *α*–*β*, *ω*–*c*_2_, and *γ*–*δ*. Within the tested simulation space, *α* and *β* are most influential for convergence speed, γ is most influential for silo-related indicators, and *ω* and *c*_2_ show a notable interaction around the baseline values. Figure 4 presents the interaction heatmaps for these parameter pairs. These findings should be interpreted as model-tuning evidence rather than direct institutional recommendations.

Extended coupling analysis reveals that *λ*_CSA_ and *fl_i_* are functionally coupled, as *λ*_CSA_ amplifies or dampens *fl_i_*’s effect. We tested a 5 by 5 grid (*λ*_CSA_ in the range 0.1 to 0.5, *fl_i_* in the range 1.0 to 3.0) with other parameters at baseline.

Table 10 reveals a non-trivial interaction, as at low *λ*_CSA_ (0.1), fli has minimal effect (30 to 32 iterations), while at high *λ*_CSA_ (0.5), *fl_i_* = 1.0 yields 36 iterations, *fl_i_* = 2.0 yields 29 (optimal), and *fl_i_* = 3.0 yields 34. The optimal combination (0.3, 2.0) yields 29 iterations, with a flat performance landscape within 3 iterations for *λ*_CSA_ in the range 0.2 to 0.4 and *fl_i_* in the range 1.5 to 2.5.

#### 5.5.1. Weight Schedule Robustness Simulation

To assess weight schedule robustness, we varied three characteristics by ±50%: transition center (baseline = 12), transition steepness (baseline = 0.5), and weight ranges for $\lambda_{GPC}$ and $\lambda_{CSA}$ (defined as $\lambda_{GPC,start}$ to $\lambda_{GPC,end}$, $\lambda_{CSA,start}$ to $\lambda_{CSA,end}$).

Table 11 reports the simulation results. The transition center shows the highest sensitivity, as shifting it to iteration 6 delays convergence to 33 iterations (Gini 0.12) and shifting it to 18 delays convergence to 32 iterations (Gini 0.11). Steepness and weight ranges show lower sensitivity, with variations within 2 iterations and 0.02 Gini. The schedule is moderately robust, with the worst case at 34 iterations versus the baseline of 29, but the transition center is a critical parameter. The fixed *λ*_CSA_ row (no transition) confirms that removing dynamic transition degrades performance (34 iterations, Gini 0.13), supporting phase-dependent weighting. Systematic hyperparameter search is identified as future research.

#### 5.5.2. Initial Distribution Robustness Simulation

To verify that performance is not an artifact of specific initial distribution, we tested five differentiated distributions. The baseline uses left-skewed (mean *Q_i_* ≈ 0.45); four additional distributions test extreme starting conditions.

The first distribution is uniform, with *Q_i_* following Uniform (0.2, 0.7), representing a heterogeneous class. The second distribution is left skewed, serving as the baseline, with *Q_i_* following Beta (2, 5) and a mean of approximately 0.29, representing a beginner-heavy class. The third distribution is right skewed, with *Q_i_* following Beta (5, 2) and a mean of approximately 0.71, representing an advanced class. The fourth distribution is bimodal, with 50 students at approximately 0.25 and 50 at approximately 0.65, representing a stratified class. The fifth distribution is clustered, with three clusters at approximately 0.20, 0.45, and 0.70. All distributions use the piecewise resistance model and baseline parameters.

Table 12 reports the robustness simulation results. The algorithm converges under all five tested distributions, requiring 27 iterations for the right-skewed distribution and 34 for the bimodal distribution. This range suggests moderate robustness to the tested initial distributions. Equity indicators are more sensitive: Gini ranges from 0.06 to 0.11, reflecting the greater difficulty of large initial gaps. The bimodal distribution is the most challenging case because the gap between modes exceeds the scaffolding zone, while the right-skewed distribution is easiest because many learners begin closer to the target threshold.

A limitation of this analysis is that the five distributions are parameterized approximations, and real classrooms may exhibit more complex distributions. The results confirm that the algorithm is not overfit to a single distribution shape, though robustness under all possible distributions cannot be guaranteed.

### 5.6. Simulated Implications and Their Empirical Validation Gaps

The simulation findings suggest model-predicted implications, each with empirical validation gaps. First, phase-dependent weighting suggests that early phases may benefit from structured hierarchical grouping, whereas later phases may benefit from peer-based error correction and social consolidation. Second, sensitivity analysis indicates that α mainly affects convergence speed and γ mainly affects distributional or network indicators within the model. Third, parameter calibration and fitness design are domain specific; direct transfer to other disciplines or institutions is not supported without recalibration.

### 5.7. Simulation–Reality Deviation Sources

This subsection analyzes deviation sources between model-predicted outcomes and real classroom outcomes. Three categories are identified.

First, even the three-dimensional competence vector is a substantial simplification. Language proficiency, note taking, and expressive delivery do not directly encode motivation, sustained engagement, learning style or strategy preference, time-varying cognitive load, interpersonal communication quality, trust, or conflict. Omitting motivation and engagement may make participation appear more consistent than it is and can, therefore, overstate convergence speed. Omitting learning style and strategy preference compresses heterogeneous responses to the same peer input, which may make stable grouping appear easier to obtain. Cognitive load is only approximated for simultaneous interpreting rather than measured as a dynamic state, so large update steps may overestimate attainable short-term gains under heavy load. Interpersonal communication is compressed into *R_ij_*, meaning that a social barrier may be incorrectly represented as a competence-gap barrier. The multi-objective extension reduces scalar masking but does not solve these omitted-variable problems.

Second, the behavioral realism gap remains substantial. The model omits or simplifies motivation, anxiety, free riding, friendship, communication style, teacher mediation, and strategic participation. Table S1 summarizes conceptual assumption grounding and the evidence needed for calibration; it is not a record of empirical validation. The resistance thresholds and their social interpretation require observed pair-level interaction data.

Third, temporal and contextual mismatch remains. The revised interpretation maps iterations to productive interaction events and restricts pedagogical interpretation to the approximately 67 events observed over 16 weeks; it no longer treats *K*_max_ = 500 as a classroom duration. Even within this horizon, learning occurs in irregular bursts, and curriculum assessments, fatigue, deadlines, and external events can alter trajectories in ways the simulator does not represent.

Addressing these gaps requires (1) incorporating time-varying motivation and affective coefficients; (2) empirically calibrating the piecewise resistance function; (3) adding free-riding and interaction-quality detection; and (4) conducting controlled classroom experiments. Until such validation is completed, the results should be treated as algorithmic design evidence.

### 5.8. Resistance Model Selection: Piecewise Main Model Compared with Monotonic Baseline

The primary piecewise, threshold-based resistance model described in Section 2.3.1 was adopted because of its alignment with ZPD logic. To evaluate this choice, we compared it with the simplified monotonic sigmoid baseline defined in Equation (3) under identical parameters (*N* = 100, *D* = 3, *K*_max_ = 500).

Table 13 reports the simulation results. The piecewise model yields 29 iterations, four slower than the monotonic baseline, reflecting additional exploration for scaffolding opportunities. However, the piecewise model improves equity-related indicators: Gini decreases from 0.12 to 0.09, CV decreases from 0.08 to 0.07, and the focused–regular gap decreases from 0.10 to 0.07. The monotonic baseline favors faster convergence, but it does not distinguish scaffolding-productive gaps from barrier-producing gaps. The piecewise model is therefore adopted as the theoretically preferred simulation setting, while its empirical calibration remains necessary.

### 5.9. Weight Scheduling Strategy Comparison

To evaluate the phase-dependent schedule against simpler alternatives, we compared (1) fixed weights (*λ*_GPC_ = *λ*_CSA_ = *λ*_PSO_ = 0.33), (2) linear varing weights, with *λ*_GPC_ decreasing from 0.5 to 0.2, *λ*_CSA_ increasing from 0.2 to 0.4, and *λ*_PSO_ increasing from 0.3 to 0.4), and (3) phase-dependent weights governed by a sigmoid transition centered at iteration 12. In all three strategies, *λ*_GPC_ + *λ*_CSA_ + *λ*_PSO_ ≈ 1.0 throughout the simulation.

Table 14 reports the simulation results. The phase-dependent schedule achieves 29 iterations with the best equity indicators among the tested schedules, while fixed weights require 42 iterations and produce higher inequality. The relatively small difference between linear and sigmoid schedules suggests that having a transition may matter more than the exact transition shape. The schedule was not optimized through exhaustive hyperparameter search, so the result should be interpreted as support for dynamic weighting rather than proof of an optimal schedule.

A robustness consideration should be noted regarding these comparisons: the results were obtained under baseline settings, and while the rankings are robust under the piecewise model, they may change under different parameter configurations. In particular, under high-resistance settings, the early GPC dominance may diminish, which could alter the relative ordering of algorithms.

## 6. Limitations

This simulation-based study operates at the model level. The initialization values are illustrative rather than verified participant-level descriptive data; the educational model and standardized repository configuration are not identical; weighted *Q_i_* is an evaluation-layer metric rather than the repository algorithms’ internal objective; the 30-run inference applies only to the standardized repository configuration; the pairwise hybrids lack repeated-run inference; and no five-fold empirical validation, qualitative triangulation, or classroom causal test is claimed. Therefore, the framework is a computational prototype requiring aligned implementation, independent replication, auditable data, and prospective ethical approval before classroom evaluation.

## 7. Conclusions and Future Research

### 7.1. Summary of Findings

This simulation-based study presents HGPC-CSA-PSO as an exploratory biomimetic framework for dynamic collaborative grouping in interpreting education. In the illustrative scalar baseline trajectory, the framework reaches the *θ* = 0.99 threshold after 29 productive interaction events, corresponding to approximately 6.9 weeks at the observed interaction rate, and therefore within the 16-week educational horizon. In the primary 30-run paired analysis, the mean convergence count is 16.367 ± 1.974 iterations. The method converges significantly faster than GPC and CSA (Holm-adjusted *p* < 0.001 for both), but the difference from PSO is not statistically significant (Holm-adjusted *p* = 0.194). The remaining baseline and pairwise-hybrid ablation values are descriptive point estimates unless repeated-run inference is explicitly reported. These are computational results under the stated assumptions, not proof of classroom effectiveness or universal optimizer superiority. Practical use requires public code/data, independent replication, and controlled empirical validation.

### 7.2. Educational and Theoretical Significance

The theoretical value of this study lies in translating selected educational constructs—peer learning, scaffolding, knowledge diffusion, and developmental equity—into an explicit simulation framework. The practical value is more limited: the model can inform how dynamic grouping might be studied computationally, but it does not yet provide a validated classroom decision tool. Because learner experience in technology-mediated settings is context dependent [35], any institutional use would require ethical review, transparent parameter calibration, teacher oversight, and empirical testing with real learners.

### 7.3. Future Research Directions

Future research should prioritize the development of a fully aligned implementation in which all mechanisms and parameters specified in the educational model are consistently operationalized, followed by repeated comparisons with standalone and two-component configurations. Ethically approved and de-identified longitudinal classroom data are also needed to calibrate the collaboration-resistance function, evaluate the empirical plausibility of the simulated trajectories, and distinguish predictive agreement from causal learning effects. Multi-site controlled studies could subsequently examine whether algorithmically supported grouping produces robust and sustained educational gains. Further research should incorporate time-varying motivation and cognitive-load states, extend the multi-objective formulation to all baseline methods, strengthen formal stochastic convergence analysis, and compare the proposed framework with adaptive optimization and reinforcement-learning-based grouping approaches using externally defined reward criteria.

## Figures and Tables

**Table 1:** Key notation and definitions for the HGPC-CSA-PSO framework.

Symbol	Definition
*X_i_*	Position of student *i* in the *D*-dimensional skill space (*D* = 3: Language proficiency *L*, Note-taking ability *N*, Expressive delivery *E*)
*Q_i_*	Proficiency level of student *i* (Equation (1))
*X*_best,*i*_	Personal best position achieved by student *i* throughout the learning process
*X*_group best_	Best position within student *i*’s current learning group
*X*_global best_	Global best position in the entire community
*d_ij_*	Euclidean distance between students *i* and *j* in the skill space
*R_ij_*	Collaboration resistance between students *i* and *j*, represented by the primary piecewise model unless otherwise stated
AP*_ij_*	Awareness probability that student *j* detects student *i*’s learning attempt (Equation (5))
*V_i_*(*t*)	Velocity (learning trajectory adjustment) of student *i* at iteration *t*
*L_i_*	Language proficiency dimension of student *i*, normalized to [0, 1]
*N_i_*	Note-taking ability dimension of student *i*, normalized to [0, 1]
*E_i_*	Expressive delivery dimension of student *i*, normalized to [0, 1]
*w*_1_, *w*_2_, *w*_3_	Weights for *L*, *N*, *E* dimensions in *Q_i_* (baseline: 0.4, 0.3, 0.3)
*ΔX_i_*	Position update (competence adjustment) of student *i*
*λ*_GPC_(*t*)	Phase-dependent weight for GPC mechanism at iteration *t* (0.5→0.2)
*λ*_CSA_(*t*)	Phase-dependent weight for CSA mechanism at iteration *t* (0.2→0.4)
*λ*_PSO_(*t*)	Phase-dependent weight for PSO mechanism at iteration *t* (0.3→0.4)
*fl_i_*	Flight length of student *i* in CSA, determining max step size (baseline: 2.0)
*δ*	Awareness coefficient modulating AP*_ij_* growth with proximity (baseline: 0.5)
*θ_m_*	Moderation threshold for CSA three-branch design, controlling width of moderate-imitation zone (baseline: 0.2)
*α*	GPC displacement coefficient (baseline: 0.7)
*β*	GPC group influence coefficient (baseline: 0.3)
*γ*	Resistance sensitivity coefficient in *R_ij_* (baseline: 0.5)
*ω*	PSO inertia weight (baseline: 0.7)
*c*_1_	PSO cognitive coefficient (baseline: 1.5)
*c*_2_	PSO social coefficient (baseline: 1.5)
*r_i_*, *r*_1_, *r*_2_	Random numbers uniformly distributed in [0, 1]
*θ*	Scalar convergence threshold for mean proficiency (baseline: 0.99)
*N*	Total number of students in the simulation (baseline: 100)
*D*	Dimensionality of the skill space (*D* = 3: *L*, *N*, *E*)
*K*_max_	Maximum number of iterations (baseline: 500)
G	Group size in GPC tier assignment
CV	Coefficient of Variation of student proficiency (distributional equity indicator)
Gini	Gini Coefficient of student proficiency (distributional equity indicator)
CC	Network Clustering Coefficient (silo suppression metric)
SHI	Structural Hole Index (inter-group connectivity metric)
BQG	Bottom-Quartile Gain: average proficiency improvement of bottom-quartile students (diagnostic lower-tail equity indicator)
ZPD	Zone of Proximal Development (Vygotsky’s framework)

**Table 2:** Multi-objective compared with scalar fitness formulation comparison (simulation-generated data, *N* = 100, *D* = 3, *K*_max_ = 500, piecewise resistance model). MO = multi-objective with MOPSO/NSGA-III; Scalar = linear weighted sum *Q_i_*.

Formulation	Convergence (Iter)	CV	Gini	BQG	Masked Students (Q ≥ 0.99 but Min Component < 0.90)	Min Component (Mean)
Scalar *Q_i_* (*w*_1_ = 0.4, *w*_2_ = 0.3, *w*_3_ = 0.3)	29	0.07	0.09	0.66	8/100	0.91
MO *F_i_* (MOPSO/NSGA-III, component-wise *θ* = 0.95)	32	0.06	0.07	0.71	0/100	0.96
Relative change	+10.3%	−14.3%	−22.2%	+7.6%	−100%	+5.5%

**Table 3:** Theory–algorithm correspondence matrix.

Theory	Principle	Algorithmic Implementation	Equation/ Parameter	Representational Fidelity
Social Cognitive Theory [29]	Observational learning	PSO velocity update toward personal/social best	Equation (6): *X*_best,*i*_, *X*_global best_	High—direct computational encoding of modeling process
Social Cognitive Theory [29]	Self-efficacy	Fitness-weighted influence term	*Q_i_* weighting in PSO	Medium—self-efficacy is multi-dimensional (confidence, persistence, affect), *Q_i_* captures only competence dimension
Social Cognitive Theory [29]	Triadic reciprocal determinism	Personal/environmental/behavioral factor mapping	*X*_best,*i*_/*R_ij_*/*ΔX_i_*	Medium—mapping is structural but does not capture bidirectional causal dynamics
Collaborative Learning Theory [13]	Positive interdependence	Group-based update terms	*X*_group best_, *X*_global best_	High—each agent’s progress tied to group performance
Collaborative Learning Theory [13]	Individual accountability	Personal best tracking	*X*_best,*i*_	High—prevents free-riding on group achievements
Collaborative Learning Theory [13]	Productive peer interaction filter	Awareness probability	AP*_ij_* (Equation (5))	Medium—models interaction blocking but not interaction quality variation
Knowledge Construction Theory [30]	Iterative knowledge synthesis	Position update as competence reconstruction	$X_{i,t+1}$ = f(*X_i_*_,*t*_, *X_j_*, *X*_group best_, *X*_global best_)	High—captures synthesis from multiple sources
Knowledge Construction Theory [30]	Community knowledge plateau	Convergence criterion	avg(*Q_i_*) ≥ *θ*	Medium—models collective convergence but not individual knowledge diversity at convergence
Gile’s Effort Model [5]	Multi-dimensional competence	Linear weighted *Q_i_*	Equation (1): *Q_i_* = *w*_1_*L_i_*+ *w*_2_*N_i_* + *w*_3_*E_i_*	Medium—linear aggregation provides scalar projection; multi-objective variant (Section Multi-Objective Fitness Formulation and MOPSO/NSGA-III Integration) addresses non-compensability
Vygotsky’s ZPD [19]	Scaffolding within reach	Collaboration resistance modulating interaction	piecewise collaboration resistance *R_ij_*	High—piecewise threshold-based formulation directly represents the specified ZPD-related resistance zones
Piaget’s Cognitive Disequilibrium [17]	Conflict-driven adjustment	CSA three-branch error-correction	Equation (4): full imitation, moderate imitation, and independent exploration branches	Medium—captures conflict→adjustment but not assimilation/accommodation distinction

**Table 4:** Grid search parameter calibration results for HGPC-CSA-PSO (simulation-generated data).

Parameter Pair	Range Tested	Optimal Value	Performance Metric
*α–β*	*α*: [0.4, 1.0], *β*: [0.1, 0.5]	*α* = 0.7, *β* = 0.3	Convergence: 29 iter
*γ*–*δ*	*γ*: [0.2, 0.8], *δ*: [0.2, 0.8]	*γ* = 0.5, *δ* = 0.5	CV: 0.07, CC: 0.17
*ω*–*c*_2_	*ω*: [0.4, 1.0], *c*_2_: [1.0, 2.0]	*ω* = 0.7, *c*_2_ = 1.5	Gini: 0.09
*c*_1_	[1.0, 2.0]	*c*_1_ = 1.5	Balanced exploration
*fl_i_*	[1.0, 3.0]	*fl_i_* = 2.0	Step size optimization
*θ*	[0.90, 0.99]	*θ* = 0.99	Convergence threshold

**Table 5:** Performance of the four algorithms over 30 independent paired simulation runs under the standardized repository configuration.

Comparison	Proposed Mean ± SD	Baseline Mean ± SD	Mean Paired Difference	95% CI	Holm-Adjusted *p*	Effect Size
HGPC-CSA-PSO vs. GPC	16.367 ± 1.974	63.567 ± 6.257	47.200	[45.067, 49.367]	<0.001	*d_z_* = 7.7459
HGPC-CSA-PSO vs. CSA	16.367 ± 1.974	30.667 ± 4.521	14.300	[12.800, 15.867]	<0.001	*d_z_* = 3.2533
HGPC-CSA-PSO vs. PSO	16.367 ± 1.974	17.133 ± 2.013	0.767	[−0.333, 1.900]	0.194	*d_z_* = 0.2427

**Table 6:** Comprehensive algorithm comparison (simulation-generated point estimates, *N* = 100, *D* = 3, *K*_max_ = 500 as a numerical safety cap). CV = Coefficient of Variation; Gini = Gini Coefficient; CC = Network Clustering Coefficient; SHI = Structural Hole Index. Lower CV, Gini, CC and higher SHI indicate better model-level outcomes. Static grouping is a fixed policy, so algorithmic convergence iterations are not applicable.

Algorithm	Convergence (Iterations)	CV	Gini	CC	SHI	Success Rate (%)
HGPC-CSA-PSO	29	0.07	0.09	0.17	0.76	90
Static Grouping	N/A (fixed policy)	0.25	0.35	0.45	0.28	45
GPC	85	0.15	0.22	0.30	0.45	68
CSA	120	0.18	0.25	0.35	0.40	62
PSO	95	0.14	0.20	0.28	0.48	70
IPSO [24]	30	0.12	0.18	0.22	0.55	82
ACO [33]	150	0.20	0.28	0.38	0.35	58
GA [21]	45	0.13	0.19	0.24	0.50	75
MOPSO [26]	35	0.11	0.16	0.20	0.58	80

**Table 7:** Convergence iteration counts for HGPC-CSA-PSO, GPC, CSA, and PSO under different student population sizes (simulation-generated data).

Student Population	HGPC-CSA-PSO	GPC	CSA	PSO
100	29	85	120	95
200	25	64	163	44
300	28	74	118	37
400	28	62	113	37
500	29	61	103	37
600	28	59	95	36
700	30	63	95	35

**Table 8:** Bottom-Quartile Gain (BQG) comparison—education-specific metric measuring average proficiency improvement of the 25 lowest-initial-proficiency students (simulation-generated data, *N* = 100, *D* = 3, *K*_max_ = 500).

Algorithm	BQG	Bottom-Quartile Final Q (Mean)	Top-Quartile Final Q (Mean)	BQ/TQ Ratio
HGPC-CSA-PSO	0.66	0.91	0.99	0.92
MOPSO [22]	0.54	0.81	0.98	0.83
IPSO [24]	0.48	0.75	0.97	0.77
GA [21]	0.41	0.68	0.96	0.71
PSO	0.35	0.62	0.95	0.65
CSA	0.38	0.65	0.94	0.69
Static Grouping	0.15	0.42	0.88	0.48

**Table 9:** Ablation experiment results (simulation-generated data). Removing any single component worsens at least one major metric, indicating that the three mechanisms contribute different functions within the hybrid framework.

Configuration	Convergence (Iterations)	CV	Gini	CC	Success Rate (%)
Full HGPC-CSA-PSO	29	0.07	0.09	0.17	90
Without GPC (CSA + PSO)	48	0.11	0.16	0.27	76
Without CSA (GPC + PSO)	43	0.10	0.14	0.24	78
Without PSO (GPC + CSA)	62	0.14	0.20	0.34	70

**Table 10:** *λ*_CSA_–*fl_i_* coupling analysis—convergence iterations for selected parameter combinations (simulation-generated data, *N* = 100, *D* = 3, *K*_max_ = 500). Baseline: *λ*_CSA_ = 0.3, *fl_i_* = 2.0.

*λ*_CSA_–*fl_i_*	1.0	1.5	2.0	2.5	3.0
0.1	32	31	30	31	32
0.2	34	31	29	30	32
0.3	36	31	29	30	33
0.4	37	32	30	31	34
0.5	36	33	29	32	34

**Table 11:** Weight schedule robustness simulation (simulation-generated data, *N* = 100, *D* = 3, *K*_max_ = 500). Baseline: transition center = 12, steepness = 0.5, *λ*_GPC_ = 0.5→0.2, *λ*_CSA_ = 0.2→0.4.

Perturbation	Convergence (Iter)	CV	Gini	CC
Baseline	29	0.07	0.09	0.17
Transition center = 6 (early)	33	0.09	0.12	0.20
Transition center = 18 (late)	32	0.08	0.11	0.19
Steepness = 0.25 (gradual)	31	0.08	0.11	0.18
Steepness = 0.75 (sharp)	30	0.07	0.09	0.17
*λ*_GPC_ range = 0.6→0.1 (wider)	31	0.08	0.11	0.18
*λ*_GPC_ range = 0.4→0.3 (narrower)	32	0.09	0.12	0.20
*λ*_CSA_ range = 0.1→0.5 (wider)	30	0.07	0.09	0.17
*λ*_CSA_ range = 0.3→0.3 (fixed)	34	0.10	0.13	0.21

**Table 12:** Initial distribution robustness simulation (simulation-generated data, *N* = 100, *D* = 3, *K*_max_ = 500, piecewise resistance model, phase-dependent weights). All metrics measured at convergence.

Initial Distribution	Mean Q_i (Init)	Convergence (Iter)	CV	Gini	BQG	Focused–Regular Gap	Success Rate (%)
Uniform (0.2–0.7)	0.45	30	0.08	0.10	0.58	0.08	88
Left-skewed Beta (2, 5) [baseline]	0.29	29	0.07	0.09	0.66	0.07	90
Right-skewed Beta (5, 2)	0.71	27	0.05	0.06	0.42	0.04	94
Bimodal (50@0.25, 50@0.65)	0.45	34	0.09	0.11	0.61	0.09	85
Clustered (3 tiers)	0.45	31	0.08	0.10	0.59	0.08	87

**Table 13:** Monotonic compared with piecewise threshold-based resistance model comparison (simulation-generated data, *N* = 100, *D* = 3, *K*_max_ = 500).

Model	Convergence (Iter)	CV	Gini	CC	SHI	Focused–Regular Gap at Convergence
Piecewise threshold-based model (primary model)	29	0.07	0.09	0.17	0.76	0.07
Monotonic sigmoid baseline (Equation (3))	25	0.08	0.12	0.15	0.72	0.10
Relative change (main compared with baseline)	+16%	−12.5%	−25%	+13.3%	+5.6%	−30%

**Table 14:** Weight scheduling strategy comparison (simulation-generated data, *N* = 100, *D* = 3, *K*_max_ = 500).

Strategy	Convergence (Iter)	CV	Gini	CC	SHI	Focused–Regular Gap
Fixed (*λ* = 0.33 each)	42	0.12	0.16	0.23	0.63	0.13
Linear transition	32	0.08	0.11	0.18	0.68	0.08
Phase-dependent (sigmoid)	29	0.07	0.09	0.17	0.76	0.07

## Data Availability

The Python implementations of HGPC-CSA-PSO and the standalone baselines, the standardized 30-run analysis script, and the associated run-level simulation outputs are available at https://github.com/YiChenxu116/HGPC-CSA-PSO/tree/master/cl2 (accessed on 12 August 2026). The repository supports reproduction of the reported 30-run results under its standardized configuration; it does not currently reproduce every conceptual educational-model extension, figure, table, or descriptive point estimate in the manuscript. No classroom-identifying data are included.

## Supplementary Materials

The following supporting information can be downloaded at: https://www.mdpi.com/article/10.3390/biomimetics11090650/s1, Table S1: Assumption grounding and realism-gap analysis for major simulation elements.
