# Supplementary material for: A Hybrid Giza Pyramids Construction–Crow Search Algorithm–Particle Swarm Optimization (HGPC-CSA-PSO) Framework for Simulating Dynamic Collaborative Grouping in Interpreting Education: A Simulation-Based Exploratory Study
Source: Biomimetics (Basel). 2026 Sep 9;11(9):650. doi: 10.3390/biomimetics11090650 (PMC13604476; doi:10.3390/biomimetics11090650)
Supplement: Supplementary file 1 [file biomimetics-11-00650-s001.zip › biomimetics-4449396-supplementary.pdf]

Table S1. Assumption grounding and realism-gap analysis for major simulation elements.

Modeled element	Conceptual grounding	Role in simulation	Evidence required to close realism gap
L, *N*, *E* competence state	Interpreting-competence literature	Defines the three-dimensional agent state	Auditable longitudinal rubric scores and reliability evidence
Interaction opportunity	Collaborative-learning literature	Motivates discrete peer-interaction updates	Time-stamped interaction records with quality and dosage measures
Peer compatibility and barriers	Scaffolding and ZPD concepts	Motivates resistance and regrouping rules	Observed pair-level interaction, trust, conflict, and support data
Learner reflection and affect	Recognized but not dynamically modeled	Defines an omitted-variable limitation	Prospective validated measures and an explicit coding protocol
Interpreting-specific demands	Task and cognitive-load theory	Motivates the educational interpretation layer	Task-level calibration and external validation data
